# A digestive allergic reaction with hypereosinophilia imputable to docetaxel in a breast cancer patient: a case report

**DOI:** 10.1186/s12885-015-2008-0

**Published:** 2015-12-21

**Authors:** Diaddin Hamdan, Christophe Leboeuf, Cathy Pereira, Nathalie Jourdan, Laurence Verneuil, Guilhem Bousquet, Anne Janin

**Affiliations:** Centre Hospitalier de Marne-la-Vallée, Service d’Oncologie Médicale, Jossigny, F-77600 France; U1165, Université Paris7, Inserm, Hôpital Saint-Louis, Paris, F-75010 France; Université Paris Diderot, Sorbonne Paris Cité, Laboratoire de Pathologie, UMR-S 1165, F-75010 Paris, France; AP-HP-Hôpital Saint-Louis, Laboratoire de Pathologie, Paris, F-75010 France; AP-HP-Hôpital Saint-Louis, Pharmacie, Paris, F-75010 France; Université de Caen Basse-Normandie, Medical School, Caen, F-14000 France; AP-HP-Hôpital Avicenne, Service d’Oncologie Médicale, Bobigny, F-93008 France; Université Paris 13, Leonard de Vinci, Villetaneuse, F-93430 France; U1165, 1 avenue Vellefaux, Paris, F-75010 France

**Keywords:** Docetaxel, Allergic reaction, Hypereosinophilia, Digestive tract

## Abstract

**Background:**

Hypereosinophilia, defined by an absolute eosinophil count of more than 1500/mm3, is rarely observed in patients treated for cancer, and rarely imputable to anti-cancer agents. Drug-induced hypereosinophilia usually appears within a few weeks of the start of treatment and resolves after discontinuation of the medication.

We report here a first case of hypereosinophilia with digestive allergic reaction imputable to docetaxel in a woman treated for breast cancer.

**Case presentation:**

This patient, with a history of childhood atopic dermatitis and asthma, underwent surgery for breast lobular carcinoma, followed with chemotherapy including 3 cycles of the FEC100 protocol and 3 cycles of docetaxel. Ten days after the second cycle of docetaxel, she had abdominal pain with diarrhea, which increased after the third cycle of docetaxel at the same dose. The blood eosinophil count increased up to 4685/mm^3^ at day 92. All biological tests were normal, except elevated seric IgE. The systematic biopsies of the upper and lower digestive tract showed diffuse edema of the lamina propria, lymphocytic infiltrate and CD117-expressing cells both in the epithelium and in the lamina propria. Electron microscopy showed a large number of degranulating mast cells, while the number of tissue eosinophils was small.

The blood eosinophil count decreased after day 96, three months after the last injection of docetaxel. After day 182, the hypereosinophilia and symptoms resolved. This spontaneous evolution, the history of atopic dermatitis and asthma, and the negativity of all biological tests performed led us to hypothesize a diagnosis of a systemic digestive Type 1 drug-induced hypersensitivity reaction. Using two validated pharmacovigilance scales, we found that docetaxel had the highest imputability score compared to the other drugs.

**Conclusion:**

Recognition of allergic reactions imputable to docetaxel is important because it requires the drug to be discontinued. In the difficult setting of anti-cancer treatment, if reintroduction of the drug is needed, a close collaboration between oncologists, gastroenterologists and allergologists is required.

**Electronic supplementary material:**

The online version of this article (doi:10.1186/s12885-015-2008-0) contains supplementary material, which is available to authorized users.

## Background

Hypereosinophilia, defined by an absolute eosinophil count of more than 1500/mm3, is rarely observed in patients treated for cancer [1]. The main drugs responsible for hyperesosinophilia are penicillins, cephalosporins, sulfas, quinolones, and non-steroid anti-inflammatory drugs [2], but hypereosinophilia is rarely imputable to anti-cancer agents (see Additional file 1: Methods M1 for details on search strategy, and Additional file 2: Table S1 for the results of the systematic literature search). Drug-induced blood hypereosinophilia usually appears within a 2 to 10 weeks of the start of treatment and resolves after discontinuation of the medication (Additional file 2: Table S1).

Blood hypereosinophilia is associated with potentially lethal clinical complications, mainly cardiac, cutaneous, neurologic or pulmonary [3].

We report here a first case of hypereosinophilia with systemic digestive allergic reaction imputable to docetaxel in a woman treated for a localized breast cancer.

## Case presentation

This 40-years-old Caucasian woman, with a history of childhood atopic dermatitis and asthma, underwent surgery for breast lobular carcinoma of 30 mm, histological grade III, expressing estrogen and progesterone receptors, with no lymph node involvement. In accordance with French guidelines, she received post-surgery chemotherapy including 3 cycles of the FEC100 protocol – 5Fluoro-Uracile 500 mg/m^2^/cycle (Accord, France), epirubicin 100 mg/m^2^/cycle (Mylan, France) cyclophosphamid 500 mg/m^2^/cycle (Baxter, France) – and 3 cycles of docetaxel 100 mg/m^2^/cycle (Docetaxel Kabi © (ATC-Code L01CD02), Fresenius, France).

**Fig. 1:**
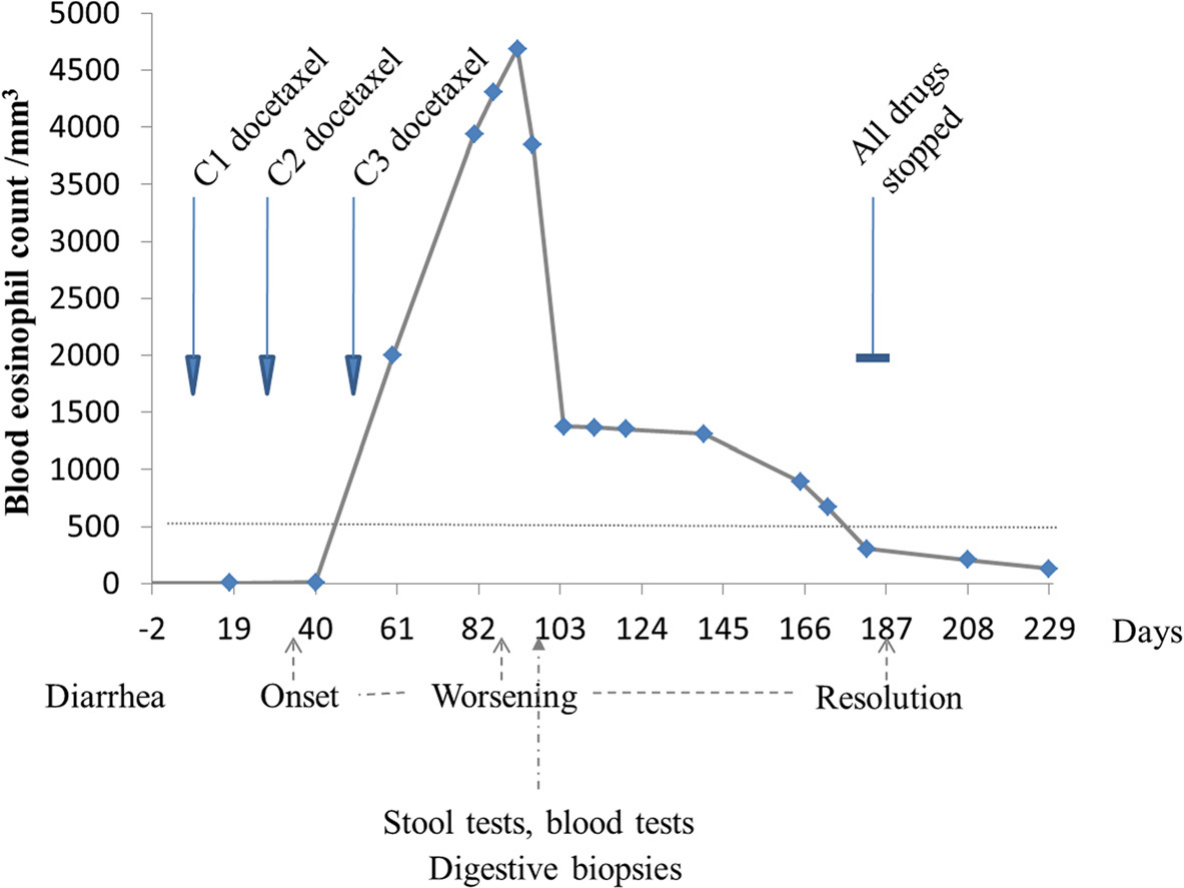
Event time-line and blood eosinophil count curve. C1, C2, C3: first, second and third cycles of docetaxel

Ten days after the second cycle of docetaxel, she had abdominal pain with diarrhea (2–5 stools/day), which increased after the third cycle of docetaxel at the same dose. The eosinophil count was 2001/mm^3^ at day 60, and 4685/mm^3^ at day 92 (Fig. 1).

**Fig. 2:**
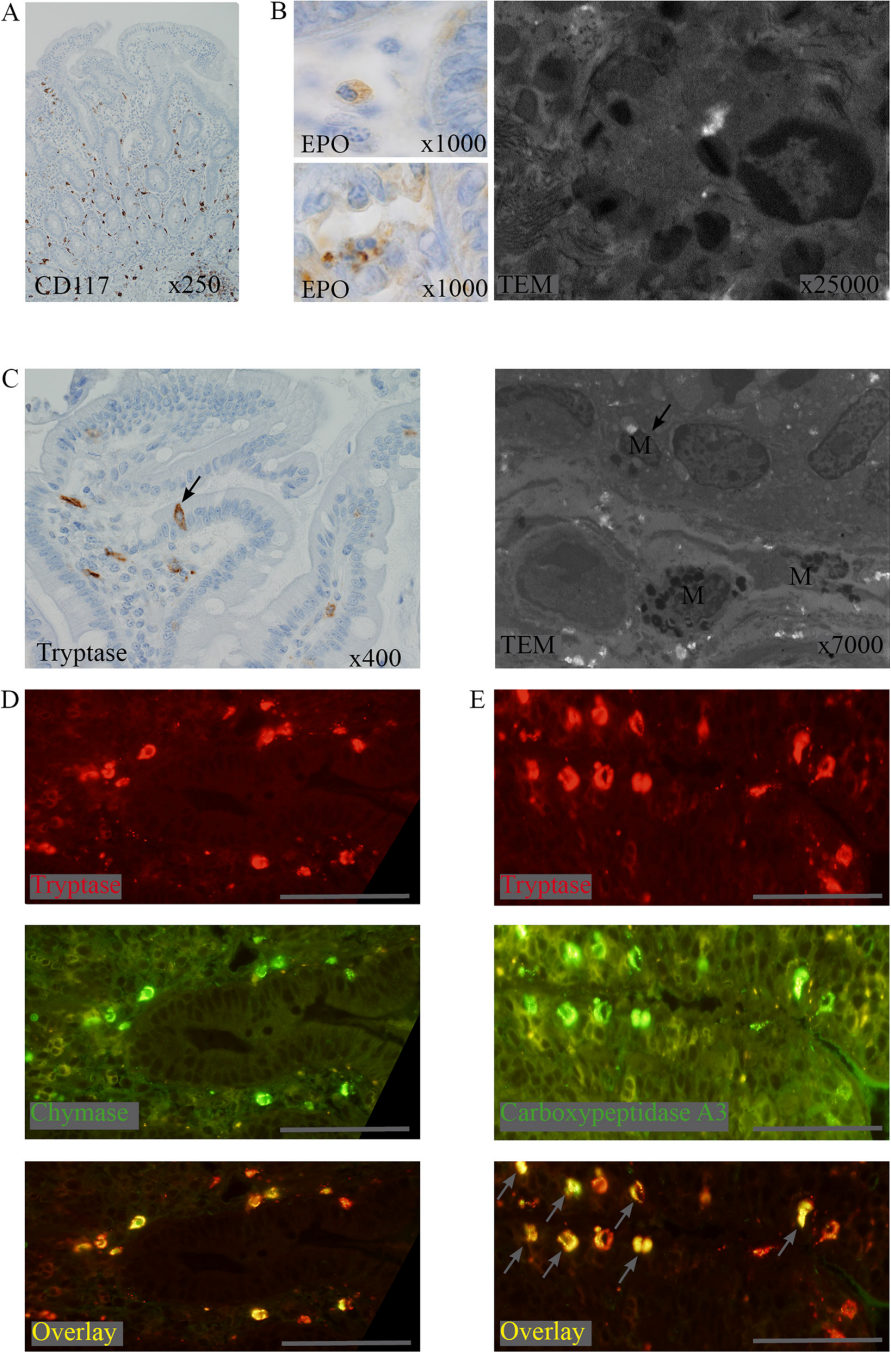
Characterization of cell infiltrates in the epithelium and lamina propria of the duodenum. Duodenal biopsies with CD117-expressing cells (**a**), which include eosinophils expressing eosinophil peroxidase (EPO) and containing specific granules (**b**); and mast cells (M) expressing tryptase and located in the lamina propria and epithelium (*arrows*, **c**). Mast cells in the lamina propria coexpress tryptase and chymase (**d**). Mast cells in the epithelium (*arrows*) coexpress tryptase and carboxypeptidase A3 (**e**)

Systematic biological tests and digestive biopsies were performed at day 92. No parasitological, bacteriological, virological, immunological or hematological cause was found; only seric IgE were elevated (Additional file 3: Table S2). The systematic biopsies of the upper and lower digestive tract showed similarities in the gut and colonic biopsies. All four biopsies had diffuse edema of the lamina propria, lymphocytic infiltrate and CD117-expressing cells both in the epithelium and in the lamina propria (see Additional file 1: Methods and Fig. 2a). We used electron microscopy, and anti-tryptase and anti-eosinophil peroxidase antibodies to differentiate and count mast cells and eosinophils in the two compartments (see Additional file 1: Methods and Fig. 2b, c, and Tables 1, 2). The diagnosis of eosinophilic gastro-enteritis was excluded because of the small number of tissue eosinophils [4]. Electron microscopy showed a large number of degranulating mast cells. No sign of thrombosis, necrosis or vascular-wall damage was found.

**Table 1:** Inflammatory cell counts in gut and colon

Digestive samples	Epithelium		
	Lymphocytes	Mast cells	Eosinophils
Duodenum	10.2 ± 4.1	4.4 ± 0.8	2.4 ± 0.7
Jejunum	12.4 ± 3.1	2.3 ± 0.5	1.2 ± 0.5
Right colon	7.8 ± 1.2	2.5 ± 0.3	0.4 ± 0.1
Left colon	8.4 ± 0.9	3.2 ± 1.1	1.3 ± 0.4

**Table 2:** Inflammatory cell counts in gut and colon

Digestive samples	Lamina propria			
	Lymphocytes	Plasma cells	Mast cells	Eosinophils
Duodenum	66.3 ± 9.2	28.7 ± 5.1	12.3 ± 2.1	4.8 ± 0.9
Jejunum	70.8 ± 10.1	26.4 ± 4.5	9.6 ± 2.5	4.2 ± 1.1
Right colon	65.5 ± 7.5	27.9 ± 5.3	8.2 ± 1.6	3.5 ± 0.6
Left colon	58.0 ± 6.7	24.3 ± 3.9	9.5 ± 1.0	2.0 ± 0.3

The blood eosinophil count decreased after day 96, three months after the last injection of docetaxel. Despite 4 months of hypereosinophilia, we did not detect cardiac, respiratory, liver or renal complications.

After day 182, the hypereosinophilia and symptoms resolved. This spontaneous evolution, the history of atopic dermatitis and asthma, and the negativity of all biological tests performed led us to hypothesize a diagnosis of a drug-induced hypersensitivity reaction (HSR). Using two validated pharmacovigilance scales, we found that docetaxel had the highest imputability score compared to the other drugs (Additional file 4: Figure S1 and Table 3).

**Table 3:** Drug imputability scores

	Adverse Drug Reaction probability scale ^a^	French imputability score ^b^			
Drugs	Score	IS	C	S	Intrinsic imputability
Docetaxel	7	2	3	3	16
Ondansetrone	4	2	1	2	12
Diosmectite	−1	2	0	1	10
Paracetamol	−1	2	0	1	10
Fluconazole	−1	2	0	1	10
Racecadotril	−1	2	0	1	10
Metoclopramide	−1	2	0	1	10
Omeprazole	−1	2	0	1	10
Phloroglucinol	−1	2	0	1	10
Prednisone	−1	2	0	1	10

*IS* Informativeness score, *C* chronology, *S* semiology

^a^Naranjo CA, Busto U, Sellers EM, et al. A method for estimating the probability of adverse drug reactions. Clin Pharmacol Ther 1981;30:239–45

^b^Arimone Y, Bidault I, Dutertre JP, et al. Updating the French method for the causality assessment of adverse drug reactions. Therapie 2013;68:69–76

Docetaxel, a semi-synthetic taxoid that inhibits depolymerization of microtubules, is currently approved for the treatment of breast, lung and prostate cancers. The most frequent adverse effects of docetaxel are hematological (pancytopenia) and digestive. Diarrhea is reported in 30 to 60 % of patients (Additional file 5: Table S3), often associated with severe oral mucositis. These toxic lesions (Type A adverse drug reaction) are predictable, dose-dependent reactions linked to prolonged or daily exposure to the drug [5], whereas HSR under docetaxel treatment (Type B immunologically-mediated adverse drug reaction according to Gell-Coombs classification, ref [5]) is dose-independent [6]. Severe HSR to docetaxel is observed in 3 % to 7.7 % of patients (Additional file 3: Table S2, and Reference [7] for review). Lethal drug-induced HSR has been reported in 0.05 % of 36,983 pati ents [8].

In the case of our patient who developed diarrhea and severe hypereosinophilia after the second injection of docetaxel, the two available pharmacovigilance scales concluded to docetaxel imputability. Since our patient had neither pancytopenia nor oral mucositis, and since docetaxel had not been administered daily or for a prolonged period, we concluded that the diarrhea was not related to a classic digestive toxicity but to Type B immune-mediated adverse drug reaction. The digestive symptoms occurred after the second injection of docetaxel, and the blood hypereosinophilia after the third injection concomitantly with elevated seric IgE. The digestive biopsies showed that the whole digestive tract was involved, with edema, large numbers of mast cells, and few eosinophils. Under electron microscopy, both eosinophils and mast cells were degranulated. Overall, these findings are in favor of a systemic digestive Type 1 hypersensitivity reaction.

Using specific antibodies (see Additional file 1: Methods), we showed that mast cells and eosinophils were distributed within the epithelium and the lamina propria. In bronchial biopsies of mild to moderate T_H_2-high asthma associated with blood eosinophilia [9], intra-epithelial mast cells co-expressed tryptase and carboxypeptidase A3, whereas mast cells of the lamina propria co-expressed tryptase and chymase. We also found these differential enzymatic co-expressions in epithelial and lamina propria mast cells in the digestive biopsies of our patient (Fig. 2d, e). In asthmatic and atopic patients, a similar immunoreactivity for IL-3, IL-5 and GM-CSF has also been found in bronchial and gut mucosa [10].

## Conclusion

We here report a case of severe HSR with hypereosinophilia imputable to docetaxel. While this condition is rare, it is important to recognize it, since it requires the drug to be discontinued. Since blood hypereosinophilia over 1500/μL and lasting more than 1 month entails a risk of major organ dysfunction [1, 11], including death through cardiac failure [12], therapy discontinuation can be recommended if these conditions are observed. In the field of adverse reactions to anti-cancer drugs, this is particularly relevant for docetaxel treatment, which can be prolonged for several months in case of good response for metastatic breast, lung or prostate cancers. If reintroduction of this anti-cancer agent is needed, a close collaboration between oncologists, gastroenterologists and allergologists is required.

## Consent

Written informed consent was obtained from the patient for publication of this Case report and any accompanying images. A copy of the written consent is available for review by the Editor of this journal.

## Additional files

Additional file 1: Methods M1. (DOCX 18 kb) Additional file 2: Table S1. Published cases of blood hypereosinophilia imputable to anti-cancer drugs. (DOCX 15 kb) Additional file 3: Table S2. Laboratory tests (DOCX 18 kb) Additional file 4: Figure S1. Drug intake between day 0 (D0) and D182. D0 is the time of the first injection of docetaxel and D182 the time when all drugs were stopped. For drugs administered continuously like omeprazole, the period of drug intake is symbolized by a straight line between the first day and the last day of treatment. (JPG 81 kb) Additional file 5: Table S3. Adverse events in clinical trials using docetaxel monotherapy. (DOCX 16 kb)

## Abbreviations

HSR: hypersensitivity reaction
