# Supplementary material for: A digestive allergic reaction with hypereosinophilia imputable to docetaxel in a breast cancer patient: a case report
Source: BMC Cancer. 2015 Dec 21;15:993. doi: 10.1186/s12885-015-2008-0 (PMC4727412; doi:10.1186/s12885-015-2008-0)
Supplement: Additional file 1: — Methods M1. (DOCX 18 kb) [file 12885_2015_2008_MOESM1_ESM.docx]

**Supplementary methods**

For the search strategy of hypereosinophilia cases imputable to anti-cancer drugs, we have applied the following method: using ad hoc algorithm composed of both thesaurus and free text terms, we searched Medline database through September 2, 2015. Design algorithm was the following:

**("Eosinophilia"[Mesh] OR "Eosinophilia" OR “eosinophilic” OR “eosinophilic syndrome” OR “hypereosinophilia”) AND ("Neoplasms"[Mesh] OR “cancer”) AND ("Drug Hypersensitivity Syndrome"[Mesh] OR "Antineoplastic Agents"[Mesh] OR "Drug Therapy"[Mesh] OR “chemotherapy” OR "drug induced")**

With the limits: Species=human and blood eosinophil count>1500/mm3, 818 articles were initially screened. Two authors (DH and GB) independently screened the papers retrieved, initially by title, then by abstract, and finally by full text.

We identified 13 publications of hypereosinophilia imputable to an anticancer agent, 10 being case reports, 2 others being Phase I clinical trials, and the last one being an observational cohort (see Supplementary Table 1).

CD117, eosinophil peroxidase (EPO) and tryptase immuno stainings were performed on 5 μm-thick tissue sections using indirect immunoperoxydase staining, with rabbit polyclonal anti-human CD117 (c-kit, Dako, Glostrup, Danemark), rabbit polyclonal anti-human EPO (ab104530, Abcam, Cambridge, UK) and monoclonal mouse-anti-human tryptase (clone G3, Santacruz, Heidelberg, Germany) as primary antibodies. Controls included omitting the primary antibody and using an irrelevant antibody of identical isotype. The analysis focused on the number and distribution of mast cells and eosinophils in the epithelial and lamina propria compartments.

For cell counts, the number of intra-epithelial lymphocytes, mast cells or eosinophils was expressed per number of 100 epithelial cells. The number of inflammatory cells in the lamina propria was expressed for a surface area of 10^4^ µm^2^ of lamina propria. Cells counts were based on an individually defined mucosal tissue unit consisting in a 4-µm-thick and 500-µm block of tissue overlaying 200 µm of muscularis mucosae [Reference 9].

Double immunofluorescent staining (tryptase/chymase, and tryptase/carbopxypeptidase A3) were performed on 5 mm-thick paraffin sections using a monoclonal mouse-anti-human mast-cell tryptase (clone G3, Santacruz, Heidelberg, Germany) and a polyclonal rabbit-anti-human chymase (CMA1, Sigma life science, Saint Louis , USA) as primary antibodies on one set of sections; the monoclonal mouse-anti-human mast-cell tryptase was used together with a polyclonal rabbit-anti-human carboxypeptidase A3 antibody (CPA3, Sigma life science, Saint Louis , USA) on another set of sections.

FITC-conjugated donkey-anti-mouse and Texas Red-conjugated donkey-anti-rabbit antibodies (both from Abcam, Cambridge, UK) were used as secondary antibodies. Controls included omitting the first antibody and using an irrelevant antibody of identical isotype.

Tissue sections were analyzed under Olympus AX 70 microscope with a 0.344-mm2 field size at × 400 magnification (Olympus, Tokyo, Japan). Images were systematically taken using SAS software, for each immunostaining image and overlay.

For electron microscopy, tissue samples were fixed in 2% glutaraldehyde-buffered 0.1 M. cacodylate, and embedded in epoxy resin. Ultra-thin sections were stained with uranyl acetate and lead citrate. Ultrastructural analysis, performed on a Hitachi-7650, focused on the epithelial and lamina propria compartments of the digestive biopsies, particularly on mucosal inflammatory cells. Images of their distribution and state of degranulation were captured.
