# Supplementary material for: A digestive allergic reaction with hypereosinophilia imputable to docetaxel in a breast cancer patient: a case report
Source: BMC Cancer. 2015 Dec 21;15:993. doi: 10.1186/s12885-015-2008-0 (PMC4727412; doi:10.1186/s12885-015-2008-0)
Supplement: Additional file 2: Table S1. — Published cases of blood hypereosinophilia imputable to anti-cancer drugs. (DOCX 15 kb) [file 12885_2015_2008_MOESM2_ESM.docx]

| **Name/class of the drug** | **Year of publication** | **Type of publication** | **First Author** | **Name of the Journal** |
| --- | --- | --- | --- | --- |
| Pan-class I PI3K inhibitor | 2015 | Phase I trial | Soria, JC | Oncologist |
| Ipilumumab/anti-CTLA4 | 2013 | Observational cohort | Voskens, CJ | PloS One |
| Lenalidomide | 2012 | Case report | Foti, C | Eur J Dermatol |
| Tosedostat/aminopeptisase inhibitor + paclitaxel | 2010 | Phase I trial | Van Herpen, CM | Br J Cancer |
| Chlorambucil | 2008 | Case report | Vaida, I | Pharmacology |
| Imatinib | 2008  2006  2005 | Case reports | Goldman, J  Le Nouail, P  Jardin, F | Ann Dermatol Venereol  Ann Dermatol Venereol  Lancet Oncol |
| Dacarbazine | 2006 | Case report | Levy, A | Ann Dermatol Venereol |
| Fludarabine | 2002  1999 | Case reports | Voutsadakis, IA  Sezer, O | Ann Hematol  Ann Hematol |
| 13-cis-retinoic acid | 1999 | Case report | Degar, BA | Med Pediatr Oncol |
| Tegafur | 1994 | Case report | Baba, M | J Gastroenterol |

Supplementary Table 1: Published cases of blood hypereosinophilia imputable to anti-cancer drugs
