# Supplementary material for: A digestive allergic reaction with hypereosinophilia imputable to docetaxel in a breast cancer patient: a case report
Source: BMC Cancer. 2015 Dec 21;15:993. doi: 10.1186/s12885-015-2008-0 (PMC4727412; doi:10.1186/s12885-015-2008-0)
Supplement: Additional file 3: Table S2. — Laboratory tests (DOCX 18 kb) [file 12885_2015_2008_MOESM3_ESM.docx]

Supplementary Table 2: Laboratory tests

| Tests | Results |
| --- | --- |
| - Parasitology |  |
| - - Stool analyses (4 successive tests) | Negative |
| - - Serology for bilharziasis, schistosmiasis, distomatosis and toxocariasis | Negative |
| - - Search in biopsy for cryptosporidium and microsporidium | Negative |
| - Immunology |  |
| - - IgE dosage | Positive 80KU/L |
| - - Complement dosage | Normal |
| - - ANCA, anti-nuclear antibodies and extractable nuclear antigen antibodies | Negative |
| - Hematology |  |
| - - Clonal T cell in blood | Negative |
| - - FIP1L1-PDGFR rearrangement | Negative |
| - Bacteriology and virology |  |
| - - Search in biopsy for salmonella, shigella, campylobacter, yersinia enterocolitica, klebsiella oxytoca and clostridium difficile | Negative |
| - - Q-PCR for cytomegalovirus, Epstein-Bar virus, herpes simplex virus 1, herpes simples virus 2, varicella zoster virus, adenovirus, enterovirus, human herpes virus 6A, 6B and 8 | Negative |

ANCA: anti-neutrophil cytoplasmic antibodies. Q-PCR: quantitative polymerase chain reaction
