# Supplementary figures and images for: A digestive allergic reaction with hypereosinophilia imputable to docetaxel in a breast cancer patient: a case report
Source: BMC Cancer. 2015 Dec 21;15:993. doi: 10.1186/s12885-015-2008-0 (PMC4727412; doi:10.1186/s12885-015-2008-0)

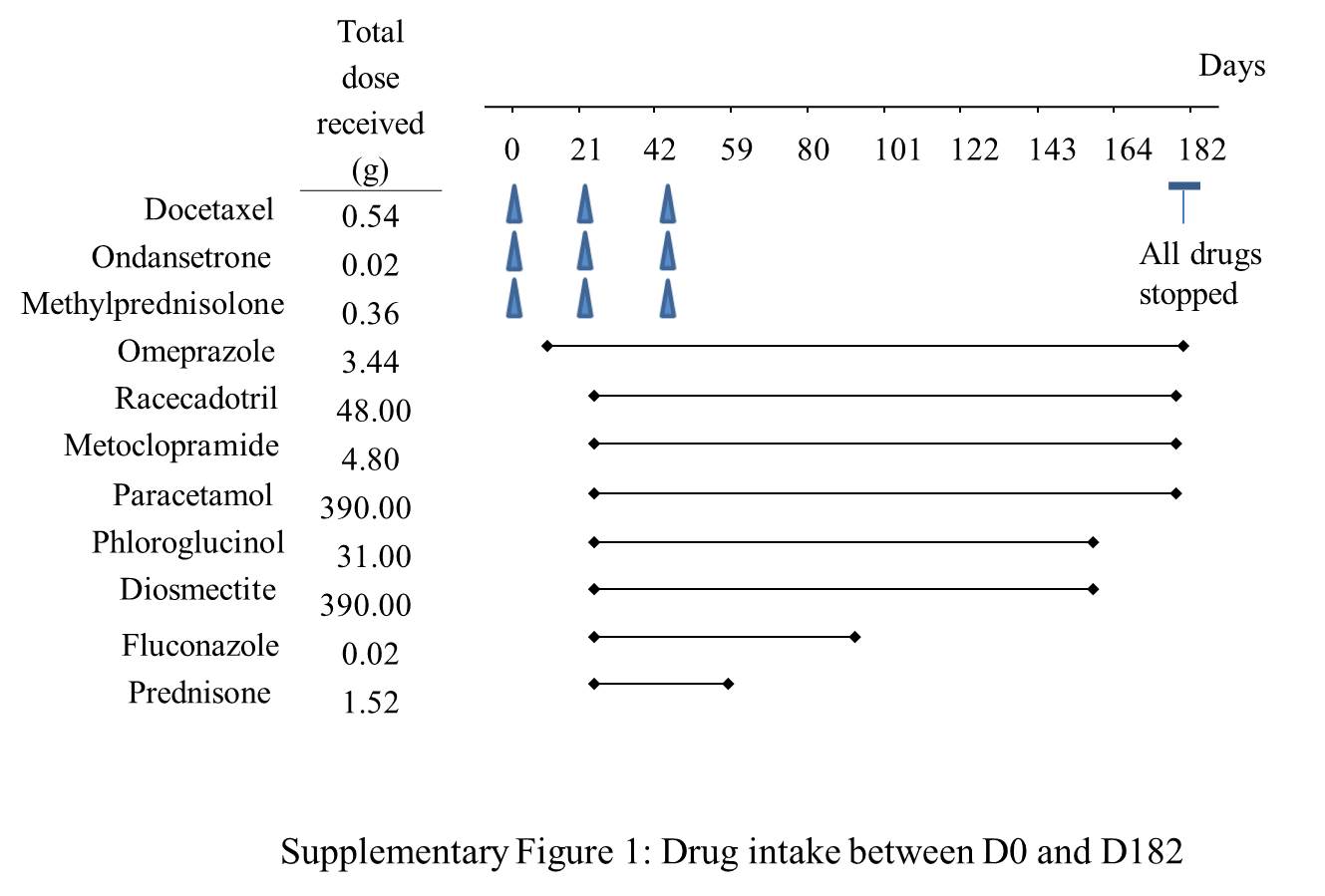

Supplement: Additional file 4: Figure S1. — Drug intake between day 0 (D0) and D182. D0 is the time of the first injection of docetaxel and D182 the time when all drugs were stopped. For drugs administered continuously like omeprazole, the period of drug intake is symbolized by a straight line between the first day and the last day of treatment. (JPG 81 kb) [file 12885_2015_2008_MOESM4_ESM.jpg]
