# Supplementary material for: A digestive allergic reaction with hypereosinophilia imputable to docetaxel in a breast cancer patient: a case report
Source: BMC Cancer. 2015 Dec 21;15:993. doi: 10.1186/s12885-015-2008-0 (PMC4727412; doi:10.1186/s12885-015-2008-0)
Supplement: Additional file 5: Table S3. — Adverse events in clinical trials using docetaxel monotherapy. (DOCX 16 kb) [file 12885_2015_2008_MOESM5_ESM.docx]

Supplementary Table 3: Adverse events in clinical trials using docetaxel monotherapy

| Trial Phase | Number of patients | Diarrhea (%) | | **Hypersensitivity reactions** (%) | | Reference |
| --- | --- | --- | --- | --- | --- | --- |
|  |  | G1-2 | G3-4 | G1-2 | **G3-4** |  |
| Phase I | 39 | NR | 5 | 2.6 | **7.7** | J Natl Cancer Inst 1992;84(23):1781-8 |
|  | 65 | 8 | 2 | 10 | **0** | Cancer Res 1993;53(5):1037-42 |
|  | 30 | 56.7 | 3.3 | NR | **3.3** | Cancer Res 1993;53(3):523-7 |
|  | 32 | 41 | NR | 18 | **6** | J Clin Oncol 1994;12(7):1458-67 |
|  | 24 | 45.8 | 8.3 | 25 | **NR** | Cancer Chemother Pharmacol 2000;45(3):213-8 |
| Phase II | 32 | NR | NR | NR | **NR** | Ann Oncol 1994;5(6):527-32 |
|  | 32 | NR | NR | NR | **3** | Eur J of Cancer 1994;30A(8):1064-7 |
|  | 34 | 38.2 | 2.9 | NR | **NR** | Eur J Cancer 1994;30A(8):1058-60 |
|  | 21 | 4 | NR | NR | **NR** | Ann Oncol 2000;11(10):1263-6 |
| Phase III | 240 | NR | 5 | NR | **NR** | J Clin Oncol 2000;18(10):2095-103 |
|  | 104 | 33 | 2.9 | NR | **NR** | J Clin Oncol2000;18(12):2354-62 |
|  | 332 | 32 | NR | NR | **NR** | N Engl J Med 2004;351(15):1502-12 |
|  | 301 | 26 | 3 | NR | **NR** | Eur J Cancer 2012;48(16):2993-3000 |
|  | 192 | 30 | <1 | NR | **NR** | Lancet Oncol 2013;14(2):149-58 |

G1-4 grades according to NCI-CTCAE

NR: not reported
